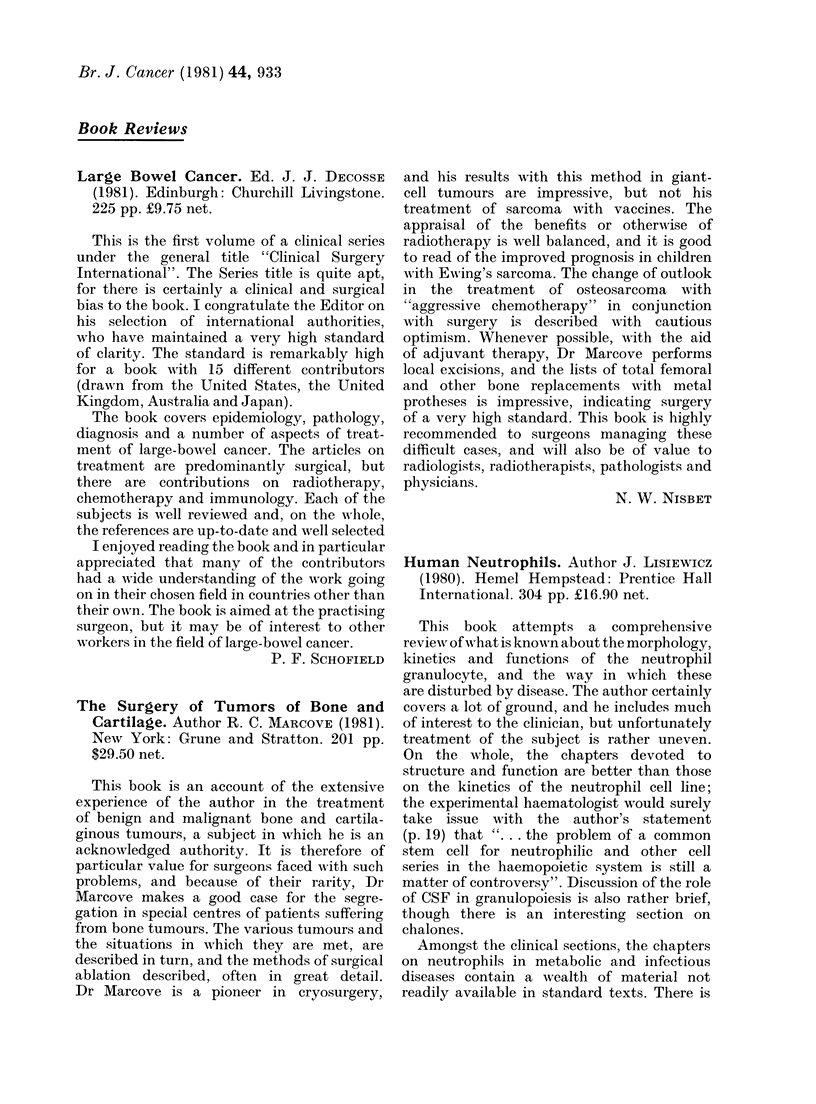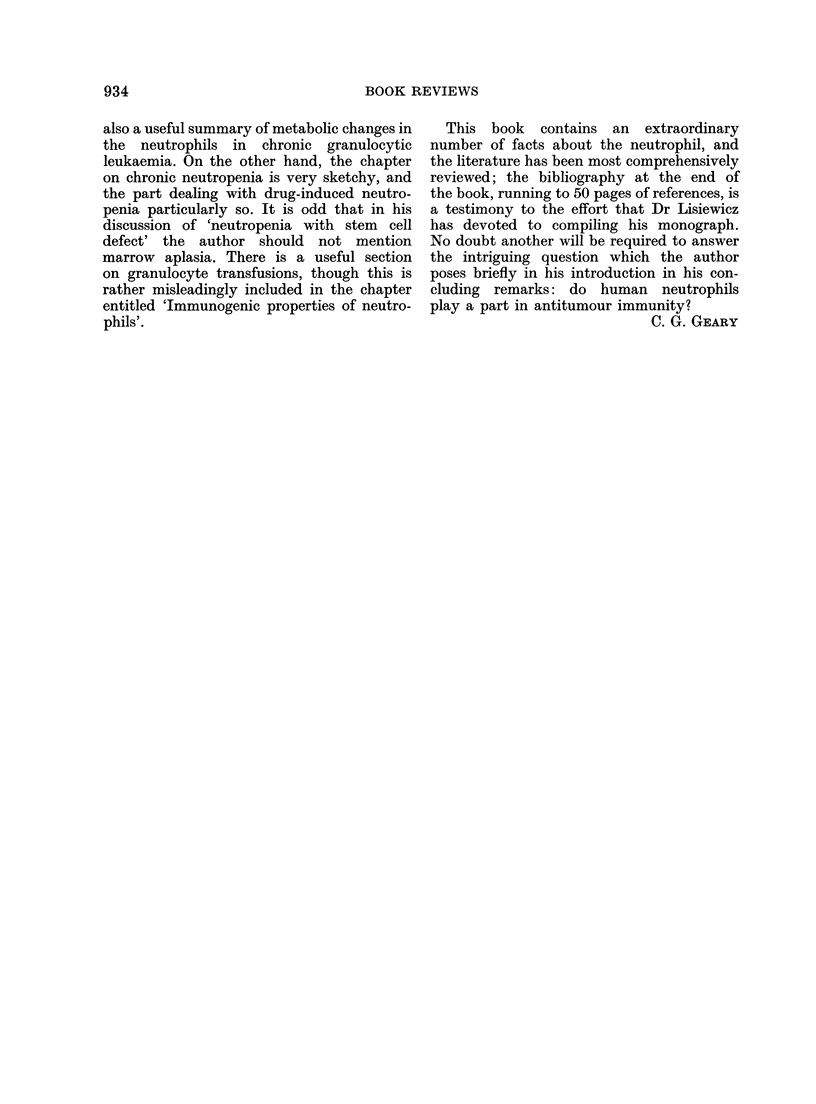# Human Neutrophils

**Published:** 1981-12

**Authors:** C. G. Geary


**Human Neutrophils.** Author J. LISIEWICZ (1980). Hemel Hempstead: Prentice Hall International. 304 pp. £16.90 net.

This book attempts a comprehensive review of what is known about the morphology, kinetics and functions of the neutrophil granulocyte, and the way in which these are disturbed by disease. The author certainly covers a lot of ground, and he includes much of interest to the clinician, but unfortunately treatment of the subject is rather uneven. On the whole, the chapters devoted to structure and function are better than those on the kinetics of the neutrophil cell line; the experimental haematologist would surely take issue with the author's statement (p. 19) that "... the problem of a common stem cell for neutrophilic and other cell series in the haemopoietic system is still a matter of controversy". Discussion of the role of CSF in granulopoiesis is also rather brief, though there is an interesting section on chalones.

Amongst the clinical sections, the chapters on neutrophils in metabolic and infectious diseases contain a wealth of material not readily available in standard texts. There is also a useful summary of metabolic changes in the neutrophils in chronic granulocytic leukaemia. On the other hand, the chapter on chronic neutropenia is very sketchy, and the part dealing with drug-induced neutropenia particularly so. It is odd that in his discussion of 'neutropenia with stem cell defect' the author should not mention marrow aplasia. There is a useful section on granulocyte transfusions, though this is rather misleadingly included in the chapter entitled 'Immunogenic properties of neutrophils'.

This book contains an extraordinary number of facts about the neutrophil, and the literature has been most comprehensively reviewed; the bibliography at the end of the book, running to 50 pages of references, is a testimony to the effort that Dr Lisiewicz has devoted to compiling his monograph. No doubt another will be required to answer the intriguing question which the author poses briefly in his introduction in his concluding remarks: do human neutrophils play a part in antitumour immunity?

C. G. GEARY